# Mitochondrial transfer between tumor and immune cells: a nexus of metabolic adaptation and immune dysfunction

**DOI:** 10.1186/s40364-026-00955-7

**Published:** 2026-06-11

**Authors:** Jianqiang Yang, Yunqi Li, Soumya Vijaya Kumar, Nabil F. Saba, Chloe Shay, Yong Teng

**Affiliations:** 1 Department of Hematology and Medical Oncology, Emory University, Atlanta, GA 30322 USA; 2 Winship Cancer Institute, Emory University, Atlanta, GA 30322 USA; 3 Wallace H. Coulter Department of Biomedical Engineering, Georgia Institute of Technology and Emory University, Atlanta, GA 30322 USA

**Keywords:** Mitochondrial transfer, TME, Tumor-stromal cell interactions, Tumor immunity, Antitumor therapy

## Abstract

The tumor microenvironment (TME) is a dynamic and highly interactive ecosystem that fuels cancer progression through coordinated cellular crosstalk. Recent studies have uncovered intercellular mitochondrial transfer as a critical adaptive mechanism within this niche. Here, we synthesize current evidence supporting a paradigm in which mitochondria function as “shared organelles”, whose bidirectional trafficking reshapes tumor and immune cell states. We discuss the mechanisms by which cancer cells acquire functional mitochondria from stromal compartments to enhance bioenergetic fitness, metabolic plasticity, and resistance to therapy. Conversely, we highlight the transfer of damaged or dysfunctional mitochondria from tumor cells to immune populations, a process that contributes to immune suppression and impaired anti-tumor responses. We further delineate the molecular and cellular networks regulating mitochondrial exchange, including tunneling nanotubes, extracellular vesicles, and cytoskeletal dynamics. Finally, we evaluate emerging therapeutic strategies aimed at disrupting mitochondrial trafficking and reprogramming TME metabolism. Collectively, this review positions mitochondrial transfer as a fundamental driver of tumor progression and a promising, yet underexplored, target for cancer therapy.

## Introduction

Mitochondria are membrane-bound organelles known as the “powerhouses” of the cell, responsible for producing energy through cellular respiration [1]. The modern understanding of mitochondria in cancer includes their roles in regulating bioenergetics, producing biosynthetic precursors, maintaining redox homeostasis, modulating calcium signaling, and controlling apoptosis. Recent studies have challenged earlier hypotheses that cancer cells rely on dysfunctional mitochondria, instead demonstrating that mitochondrial reprogramming, including shifts in function and structural reorganization, is essential for tumor growth, survival, and metastasis [1, 2]. In cancer cells, mitochondria act as metabolic hubs that integrate nutrient availability with the demands of rapid proliferation. While mitochondrial reactive oxygen species (ROS) were historically viewed as the primary link between mitochondrial dysfunction and tumorigenesis, it is now clear that mitochondrial contributions to cancer extend far beyond oxidative damage. Beyond their role in ROS signaling, mitochondria directly contribute to cancer progression through genetic and epigenetic regulation. The mitochondrial electron transport chain (ETC), particularly complex III-mediated ubiquinol oxidation, promotes tumor growth by regenerating NAD⁺ and FAD, and by supporting pyrimidine biosynthesis via dihydroorotate dehydrogenase [3]. Moreover, mitochondrial-derived metabolites influence epigenetic regulation. Central TCA cycle intermediates, α-ketoglutarate (α-KG), succinate, fumarate, acetyl-CoA, and NAD^+^, serve as cofactors or inhibitors for chromatin-modifying enzymes (e.g., α-KG-dependent dioxygenases and histone acetyltransferases), linking mitochondrial metabolism to changes in DNA methylation and histone modifications that drive cancer cell phenotype [4].

Cancer cells undergo extensive structural reorganization of their mitochondrial networks to support increased bioenergetic and biosynthetic demands [5, 6]. One emerging mechanism contributing to cancer progression and therapy resistance is intercellular mitochondrial transfer (IMT), also known as horizontal mitochondrial transfer (HMT), the movement of intact mitochondria or mtDNA between cells [7]. IMT was first demonstrated by the finding that mesenchymal stem cells (MSCs) could transfer defective mitochondria to mitochondria-deficient A549 lung cancer cells, rescuing their aerobic respiration [8]. IMT is now recognized as a process regulated by cancer cells to enhance metabolic plasticity and metastatic potential, adapt to hostile microenvironments, evade immune surveillance, and contribute to therapy resistance. For example, cancer-associated fibroblasts (CAFs) can donate mitochondria to malignant cells, boosting their migratory and invasive capabilities [9]. These insights position IMT as a promising and underexplored aspect of tumor biology that may offer important therapeutic implications. A recent study demonstrated that IMT enables cancer cells to escape immune surveillance, thereby reshaping tumor-immune interactions [10]. In addition to immune cells surrounding tumor cells, various stromal cells, including astrocytes, mesenchymal stem cells (MSCs), and endothelial cells (ECs), have been shown to donate mitochondria to cancer cells [11–13]. This transfer restores oxidative phosphorylation (OXPHOS) capacity, promotes chemoresistance, and fuels metastatic dissemination [14, 15]. Interestingly, while acquiring mitochondria enhances tumor cell survival, it may also introduce metabolic vulnerabilities. For example, mitochondria transferred from MSCs can increase the chemoresistance of glioblastoma stem-like cells (GSCs), a phenomenon that could be exploited for novel therapeutic strategies [16].

Mitochondria are now viewed as shared resources that mediate intercellular communication among resident cells in the TME. This review examines the emerging significance of IMT in the TME, emphasizing a paradigm shift in our understanding of mitochondrial function as a dynamic, intercellular process. We analyze the triggers and downstream consequences of IMT and consider its impact on cancer progression and therapy resistance, alongside potential mitochondrial-targeted therapeutic approaches in oncology.

## Mechanisms of IMT

**Fig. 1:**
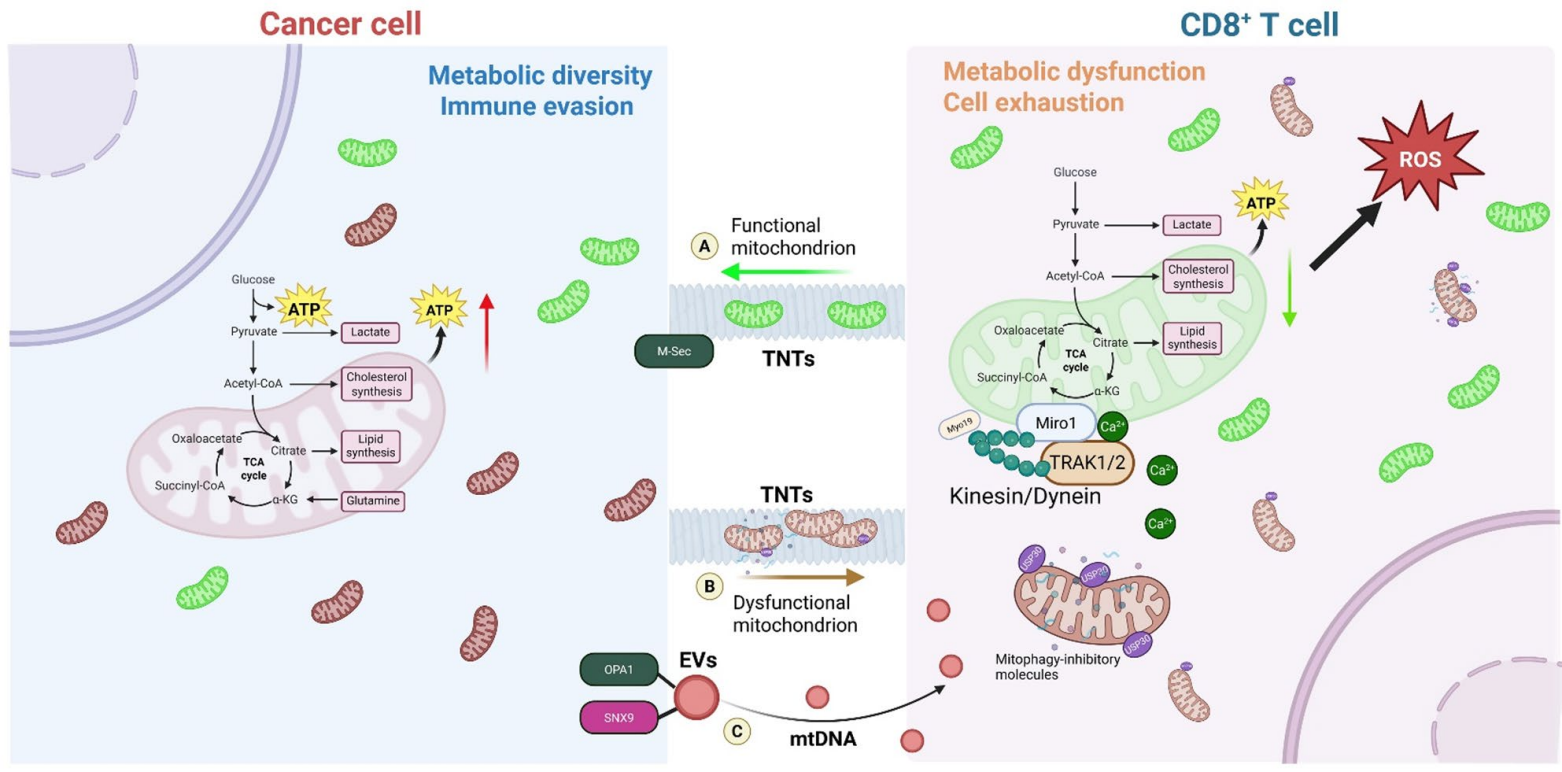
Cancer cells orchestrate bidirectional mitochondrial exchange to promote immune evasion via TNTs and EVs. (**A**) The acquisition of T cell-derived mitochondria enhances tumor aggressiveness (right to left). Cancer cells actively obtain functional mitochondria (green) from CD8⁺ T cells through tunneling nanotubes (TNTs), a process mediated by microtubule motor proteins, kinesin (anterograde) and dynein (retrograde), via the mitochondrial Rho GTPase Miro1 and its adaptors TRAK1/2. (**B**) Cancer cells impair T cell-mediated antitumor immunity through mitochondrial sabotage (left to right), transferring dysfunctional mitochondria (red) into CD8⁺ T cells via TNTs and disrupting bioenergetics and reducing metabolic fitness and effector function. Mechanistically, this process is reinforced by the co-transfer of USP30 or other mitophagy-inhibitory molecules, which prevent the clearance of damaged mitochondria and exacerbate T cell dysfunction. (**C**) Cancer cells package mitochondrial DNA (mtDNA) into extracellular vesicles (EVs; red spheres), which are subsequently internalized by CD8⁺ T cells (left to right). This process is governed by the OPA1-SNX9 axis, wherein oligomeric inner-membrane OPA1 facilitates the sorting of functional mitochondrial cargo and cytosolic SNX9 promotes vesicle scission and EV biogenesis, ultimately driving T cell exhaustion, functional impairment, and metabolic reprogramming. Figure created using Biorender (https://biorender.com)

IMT proceeds via a spectrum of mechanisms that permit mitochondria to move between cells. These mechanisms include physical conduits such as tunneling nanotubes (TNTs), vesicular trafficking via extracellular vesicles (EVs) (Fig. 1), direct intercellular interactions through gap junctions or cell fusion. Each mechanism contributes differently to the exchange of mitochondria within the TME, and these processes are associated with changes in cancer cell survival, adaptation, and immune modulation (Table 1).

**Table 1:** Key molecules involved in mitochondrial transfer signaling

Molecule	Role	Mechanism	Ref
Miro1	Anchors mitochondria to motor proteins	Connects the mitochondrial adaptor MIRO with the motor adaptor TRAK	[17–19]
Miro2	Regulates mitochondrial positioning and transport	Controls mitochondrial fission and supports retrograde mitochondrial trafficking	[20]
TRAK1/2	TRAK1 promotes mitochondrial transport into TNTs, whereas TRAK2 regulates mitochondrial distribution and short-range transport	TRAK1 couples mitochondria to both kinesin and dynein motors; TRAK2 primarily interacts with dynein	[21, 22]
F-actin	Anchor mitochondria to the cytoskeleton	Forms the structural backbone of TNTs	[23]
CD38	Regulates TNT formation	Mediates calcium-dependent signaling pathways	[24]
Epimedin C	Promotes F-actin assembly	Enhances polymerization of G-actin into F-actin through downregulation of Tβ4	[25]
Connexin 43	Functions as a TNT communicator	Forms gap junctions within TNTs to facilitate intercellular signaling	[26–28]
ICAM-1	Mediates mitochondrial transfer	Promotes cell-cell adhesion and TNT formation	[29, 30]
Myo-19	Facilitates mitochondrial movement along actin filaments	Links mitochondria with actin and ER networks	[31, 32]
OPA1/SNX9	Facilitates selective incorporation of mitochondrial components into EVs	Regulates mitochondrial-derived vesicle (MDV) formation for cargo packaging	[33, 34]

### Main mechanisms of IMT

#### IMT through TNTs

TNTs are dynamic, actin-driven membranous channels that connect distant cells, enabling the long-range, bidirectional transfer of cytoplasmic components [35] and thus contributing to tissue homeostasis under physiological conditions. Structurally, TNTs are heterogeneous and can be broadly classified into two types: thin TNTs (diameter < 0.7 μm), which contain only F-actin and transport small cargo, and thick TNTs (diameter > 0.7 μm), which incorporate both F-actin and microtubules, providing the rigidity needed for long-distance transport of large organelles such as mitochondria [36]. The biogenesis of TNTs is regulated by actin cytoskeletal remodeling and various signaling pathways. Key regulators include the small GTPases Cdc42 and Rac1, as well as the protein M-Sec (TNFaip2), which interacts with RalA and the exocyst complex to initiate membrane protrusion. In ECs, examples such as both wheat germ agglutinin (WGA) and thrombin were proven to induce TNT biogenesis [37]. A different set of mechanisms drives TNT biogenesis in cancer cells, involving the activation of epidermal growth factor receptor (EGFR) signaling, which in turn triggers downstream mitogen-activated protein kinase (MAPK) cascades to promote TNT formation [38].

IMT through TNTs has been documented in several tumor types and is considered especially significant in the TME based on emerging evidence [11]. In bladder cancer, for example, TNT-initiated mitochondria transfer enhances cancer cell invasion and metastatic potential [39]. Cancer cells exhibit specific adaptations to optimize this process. The mitochondrial Rho GTPase Miro1, which acts as a calcium-sensitive motor adaptor protein, is a key enhancer of mitochondrial transport along TNTs, particularly under metabolic stress [40, 41]. Accumulating evidence supports a role for TNT-mediated mitochondrial transfer in restoring OXPHOS in recipient cells, promoting chemoresistance, enabling metabolic flexibility, and inducing T-cell senescence to facilitate immune evasion [34, 42, 43]. These multifaceted roles firmly position the inhibition of TNT formation and function as an emerging therapeutic strategy.

#### IMT through EVs

Beyond TNTs, mitochondria and their components are efficiently trafficked via diverse EVs, including exosomes (originating from multivesicular bodies), microvesicles (which bud from the plasma membrane), and MDVs. MDVs are a subset of vesicles that originate specifically from mitochondria and selectively package damaged or functional mitochondrial proteins for secretion, thus contributing to mitochondrial quality control while preventing excessive release of mitochondrial damage-associated molecular patterns (DAMPs) [44]. MDV formation is regulated by mitochondrial fusion protein OPA1 and sorting nexins such as Snx9, which orchestrate selective budding of vesicles [44].

Encapsulation within EVs allows lipid bilayers to provide a critical layer of protection, shielding the mitochondrial cargo from extracellular enzymatic degradation and ensuring its functional delivery to recipient cells [45, 46]. This mechanism is vital for intercellular organelle exchange. An example is the transfer of mitochondria from astrocytes to neurons after ischemic stroke, which enhances neuronal survival by restoring bioenergetics [47]. Similarly, the role of EV-mediated mitochondrial transfer is complex and critical for progression. A landmark study revealed that cancer cells can package mitochondria with mutated mtDNA into EVs and transfer to tumor-infiltrating T cells (TILs), delivering not only dysfunctional organelles but also mitophagy-inhibitory molecules [10]. This two-pronged process drives homoplasmic replacement of the T cell mitochondrial network, resulting in metabolic insufficiency, induction of senescence, and markedly impaired antitumor immunity.

#### IMT through gap junctions and cell fusion

IMT also occurs through direct cytoplasmic continuity established by gap junctions or cell fusion. Gap junctions, formed by connexins such as connexin 43 (CX43), can provide intercellular communication channels that permit the passage of ions, metabolites, and potentially mitochondrial fragments [48]. Gap junction channel gating is regulated by CO₂ levels and voltage changes, which modulate the extent of intercellular connectivity [49]. For example, CX43 was demonstrated to facilitate mitochondrial transfer from bone marrow stromal cells (BMSCs) to leukemia stem cells (LSCs) [50]. This transfer promoted cancer cell metabolic function, survival, and stemness-associated gene expression programs. In addition to partial transfer via connexin channels, complete mitochondrial transfer has been observed during cell fusion. For example, fusion between MSCs and ischemic cardiomyoblasts facilitated full mitochondrial exchange, supporting metabolic recovery in recipient cells [51].

### Mechanisms governing IMT

Recent evidence challenges the notion that IMT is a stochastic process, revealing instead a highly selective and regulated system. The selective transfer of dysfunctional mitochondria to immune cells is orchestrated by specific molecular “passports” and hijacking of recipient quality control. It has been reported that cancer cells selectively enrich their mitochondria with USP30, a deubiquitinase anchored to the outer mitochondrial membrane through its N-terminal transmembrane domain [10]. USP30 continuously removes ubiquitin chains from mitochondrial surface proteins, thereby antagonizing Parkin-mediated ubiquitination and suppressing mitophagy. This mechanism enables tumor-derived mitochondria to evade PINK1/Parkin-dependent autophagic clearance prior to intercellular transfer. Functional validation studies further demonstrated that USP30 is co-transferred with mitochondria into recipient CD8⁺ T cells. Importantly, CRISPR-mediated deletion of USP30 in donor tumor cells abolished the persistence of transferred mitochondria in recipient T cells. These mitochondria became rapidly ubiquitinated, recruited Parkin, and underwent mitophagic degradation. Consequently, USP30-deficient tumor mitochondria failed to integrate into the T-cell mitochondrial network or induce T-cell exhaustion, whereas re-expression of USP30 fully restored their immunosuppressive function [10]. These findings establish USP30 co-transfer as both necessary and sufficient for the long-term survival and immunosuppressive activity of tumor-derived mitochondria within T cells. Selectivity is further regulated at the level of mitochondrial trafficking and motility. The mitochondrial Rho GTPase Miro1 acts as a molecular switch that determines the fate of mitochondria under stress. In response to calcium influx or ROS, Miro1 dissociates from microtubule-based kinesin motors and engages myosin XIX, facilitating the redirection of mitochondria into actin-rich TNTs for intercellular export [52]. This motor-switching mechanism ensures that only mitochondria mobilized by specific stress signals are selected for transfer.

Furthermore, emerging evidence suggests that cancer cells act as central “redistribution hubs” rather than mere recipients or donors. A recent study demonstrated that cancer cells acquire mitochondria from host cells, fuse them into their endogenous network, a process that enhances the filamentous assembly of P5CS to boost biosynthetic capacity, and subsequently redistribute these refurbished organelles to immunosuppressive neutrophils, macrophages, and CD4^+^ T cells, thereby orchestrating a pro-tumor ecosystem while driving CD8^+^ T cell exhaustion [53]. This paradigm reframes IMT as an active organizational process wherein cancer cells exert control over the metabolic landscape by selectively redistributing mitochondrial resources.

## Donor and recipient cells during IMT

**Fig. 2:**
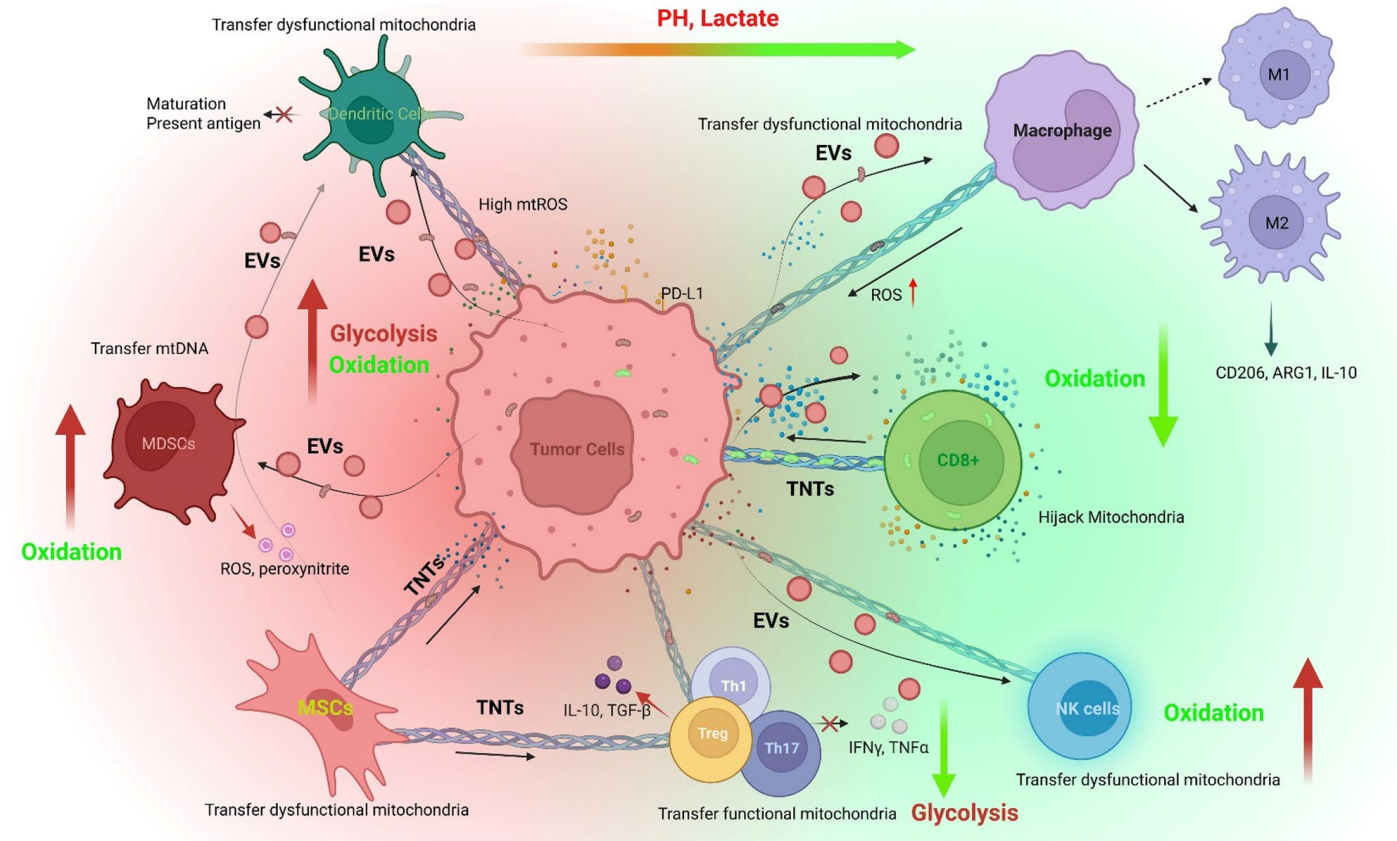
Mitochondrial transfer in the tumor microenvironment reprograms cellular metabolism and immune landscape. CD8⁺ T cells or MSCs, inadvertently donate functional mitochondria to cancer cells, a “hijacking” that enhances tumor OXPHOS and supports survival. Conversely, cancer cells release dysfunctional mitochondria enriched in ROS and mtDNA into effector immune cells such as NK cells and macrophages cells, impairing cytotoxicity and cytokine production. Cancer cells can also transfer functional mitochondria to immunosuppressive populations, including MDSCs and Tregs, promoting their proliferation and reinforcing immunosuppressive phenotypes. Additionally, mitochondrial transfer can polarize macrophages toward a pro-tumorigenic M2 state, characterized by elevated OXPHOS and upregulation of CD206, ARG1, and IL-10. Figure created using Biorender (https://biorender.com)

The exchange of mitochondria in the TME is not a random event but a highly orchestrated process involving specific donor and recipient cells (Fig. 2). Cancer cells act as both thieves and donors, reprogramming their surroundings through organelle exchange (Table 2). Cellular crosstalk creates a network of metabolic support that is fundamental to tumor progression.

**Table 2:** Representative studies of intercellular mitochondrial transfer in cancer

Cancer type	Donor cells	Recipient cells	Transfer mechanism	Functional consequence	Ref
Lung cancer	CD8^+^ T cells	Cancer cells	TNTs	Enhances OXPHOS and supports cancer cell survival	[50]
Glioblastoma	Astrocytes	Cancer cells	TNTs	Increases OXPHOS, promoting tumor proliferation and tumorigenicity	[12]
Breast cancer	Neuron	Cancer cells	Cell to cell junction	Enhances metabolic capacity, stemness, and metastatic potential	[54]
Melanoma	MSCs	CD8^+^ T cells	TNTs	Improves T cell metabolic fitness, expansion, and antitumor activity	[15]
Breast cancer	MSCs	CD4^+^ T cells	TNTs	Suppresses Th1 differentiation and effector function	[55]
Multiple cancers	Macrophages	Cancer cells	Cell to cell junction	Activates ERK signaling to promote tumor proliferation	[56]
Breast cancer	Adipose stem cells	Cancer cells	TNTs	Enhances mitochondrial activity and induces multidrug resistance	[57]
Breast cancer	CAFs	Cancer cells	TNTs	Increases OXPHOS and supports metabolic adaptation	[9]
Squamous cell carcinoma	Cancer cells	CAFs	TNTs	Reprograms fibroblast metabolism and promotes pro-tumorigenic secretome and matrisome remodeling	[20]
Multiple cancers	Cancer cells	Tumor-infiltrating lymphocytes	TNTs and EVs	Transfers mutated mtDNA, inducing T cell metabolic dysfunction and impaired antitumor immunity	[10]
Glioblastoma	Cancer cells	Astrocytes	EVs	Enhances mitochondrial respiration in astrocytes	[58]

### IMT between tumor and CD8^+^ T cells

Mitochondrial fitness is a critical determinant of CD8⁺ T cell fate and function within the TME. For CD8⁺ T cells to effectively differentiate into cytotoxic lymphocytes (CTLs) and mount an anti-tumor response, they require functional mitochondria to meet immense bioenergetic and biosynthetic demands [59–61]. Mitochondrial fitness is severely compromised through two primary mechanisms: internal metabolic reprogramming and mitochondrial hijacking by cancer cells. The tumor killing ability is impaired by the TOX expression and decreased IFN-γ expression and by the ROS-generating mitochondria in the CD8⁺ T cells, reprogramming them to an exhausted phenotype [62]. This exhaustion program is orchestrated by a network of transcription factors, including TOX, NFAT, and IRF4, that are sensitive to mitochondrial redox status and metabolic cues. When mitochondrial function deteriorates, the resulting increase in mitochondrial ROS and depletion of α‑ketoglutarate can stabilize HIF‑1α and drive the expression of exhaustion‑associated genes, linking organelle fitness directly to transcriptional fate decisions [63, 64].

Enhancing mitochondrial quality can restore CD8⁺ T-cell function. CD28-driven metabolic reprogramming improves mitochondrial mass, promotes mitochondrial fusion, increases mitochondrial membrane potential (Δψm), and enhances OXPHOS capacity, thereby restoring antitumor activity and highlighting a promising therapeutic strategy [42, 65]. Notably, the beneficial effect of enhanced mitochondrial fitness on T cell function is mediated in part through metabolic checkpoints: AMPK and mTOR coordinate nutrient sensing with mitochondrial biogenesis, while the transcription factor PGC‑1α integrates mitochondrial health with the epigenetic landscape by modulating histone acetylation and DNA methylation at effector gene loci [64, 66, 67]. Beyond intrinsic metabolic defects, mitochondrial fitness in CD8⁺ T cells is further impaired through direct bidirectional mitochondrial exchange with tumor cells. RNA-seq analyses have revealed predominantly unidirectional mitochondrial transfer from CD8⁺ T cells to tumor cells, whereby the donated mitochondria enhance tumor OXPHOS while depleting T cells of functional mitochondria and promoting exhaustion [68]. Through this mitochondrial hijacking process, cancer cells augment their bioenergetic capacity, whereas mitochondrial loss in T cells drives dysfunction and establishes a non-responsive antitumor immune state (Fig. 1). Conversely, tumor cells can transfer their own mitochondria into CD8⁺ T cells. This process has been visualized in co-culture systems using fluorescently labeled mitochondrial proteins and further validated in murine models by detecting labeled tumor-derived mitochondria within tumor-infiltrating T cells [10, 68]. Notably, this bidirectional exchange follows a distinct temporal sequence: tumor cells initially acquire mitochondria from T cells during an early “metabolic exploitation” phase, and after approximately 24 h of co-culture, they subsequently deliver their own mitochondria into T cells during a later “immunosuppressive” phase [51]. This was validated by detecting labeled tumor mitochondria within tumor-infiltrating T cells of mouse models [10]. Crucially, the transferred tumor-derived mitochondria are dysfunctional, harboring mutated mtDNA and co-transferring the deubiquitinase USP30, as discuss above, which enables them to evade PINK1/Parkin-mediated mitophagy in recipient T cells [10]. Once internalized by CD8⁺ T cells, these dysfunctional mitochondria disrupt cellular metabolism, promote T-cell exhaustion, and ultimately impair antitumor immunity [10, 51].

### IMT between tumor and CD4⁺ T cells

Mitochondrial integrity and metabolic fitness are fundamental for CD4^+^ T cell mediated antitumor immunity [69]. CD4^+^ T cells, including Th1 effectors and regulatory T cells (Tregs), rely on oxidative phosphorylation and proper mitochondrial dynamics to sustain proliferation, cytokine production, and lineage stability. In particular, the suppressive function of Tregs is tightly linked to mitochondrial metabolism and Foxp3 expression, while helper CD4^+^ T cells require intact mitochondrial signaling for effective activation and memory formation [70]. Disruption of mitochondrial quality through ROS overproduction, calcium dysregulation, or mtDNA damage-impairs CD4^+^ T cell function and promotes immune evasion within the tumor microenvironment [71, 72].

A recent study by Terasaki et al. has uncovered that cancer cells import host-derived mitochondria, integrate them into their endogenous network, and subsequently transfer them to neighboring immune cells, including CD4^+^ T cells, which results in highly suppressive states and drives CD8⁺ T cell exhaustion [53]. Importantly, they also found that the incoming mitochondria can induce filamentous P5CS assembly in cancer cells, which enables the refurbishment of damaged mitochondria into fully functional units. As a result, disrupting mitochondrial redistribution collapses the immunosuppressive ecosystem and impairs tumor growth [53]. Unlike previously studied mitochondrial exchange between cancer cells and CD8^+^ T cells, the functional consequences of mitochondrial donation from cancer cells to CD4^+^ T cells remain largely unknown. Key questions include how transferred mitochondria affect CD4^+^ subset differentiation (Th1/Treg balance), whether healthy vs. damaged mitochondria produce opposing outcomes, and if blocking this transfer can restore CD4^+^ mediated anti-tumor responses. Strategic manipulation of this pathway, either by preventing pathological mitochondrial hijacking or by engineering healthy mitochondrial delivery, holds promise for next generation cancer immunotherapy targeting CD4^+^ T cell metabolic reprogramming.

### IMT between tumor and other immune cells

Apart from T cells, cancer cells also engage in complex mitochondrial crosstalk with other immune cells, including macrophages, natural killer (NK) cells, and myeloid‑derived suppressor cells (MDSCs), to remodel the TME and suppress antitumor immunity.

Macrophages in the TME are also engaged in the mitochondrial transfer process, with emerging evidence indicating immunomodulatory consequences [53]. Hepatocellular carcinoma (HCC) cells can offload damaged mitochondria to macrophages through EVs for survival, which dispose of their “toxic waste” to evade immune detection [73]. Another study has shown that macrophages can donate their dysfunctional mitochondria to cancer cells and promote cancer cell proliferation [56]. The presence of fragmented mitochondria in tumor‑associated macrophages (TAMs) correlates with increased ROS within cancer cells, which has been proposed to contribute to activation of signaling pathways that facilitate tumor progression and therapy resistance [13, 74, 75]. Similarly, transfer of ROS‑producing mitochondria from TAMs has been linked to elevated oxidative stress in cancer cells, with some evidence pointing to activation of ERK signaling and increased invasive behavior. Although these observations suggest a reciprocal metabolic interplay between tumors and macrophages, direct in vivo validation of the directionality and functional consequences of mitochondrial exchange remains limited. Tumors can reprogram macrophages toward a tolerogenic state, while macrophage mitochondria may reciprocally influence tumor metabolism. It is also hypothesized that IMT contributes to alterations in macrophage polarization (e.g., M1 versus M2 phenotypes), which could influence the balance between antitumor and pro‑tumor immune responses. However, definitive evidence demonstrating a causative role for mitochondrial transfer in driving specific polarization states in vivo is still needed [75].

NK cells rapidly lose functionality upon entering the tumor microenvironment due to metabolic stress, mitochondrial fragmentation, and reduced respiratory capacity, resulting in impaired cytotoxicity and diminished cytokine production [76]. One study demonstrated that cancer cells hijack mitochondria from a wide array of immune cells, including NK cells, and the loss of mitochondria from NK cells is shown to reduce their activation and cytotoxic capacity against tumors [77]. Mitochondrial dysfunction and oxidative stress drive NK cell exhaustion, which is therapeutically reversible through the external donation of healthy mitochondria [78]. Such mitochondrial supplementation enhances NK cell mitochondrial fitness, promoting proliferation and improving tumor cell killing [68, 79]. Exhausted NK cells display metabolic dysregulation, including reduced OXPHOS and ATP production, as well as upregulation of inhibitory receptors such as PD-1 and TIGIT [80]. In addition, metabolic stress disrupts key signaling pathways essential for NK function, including the IRE1α-XBP1s-MYC axis, further accelerating functional collapse [81]. To date, while the field has established that NK cells are victims of mitochondrial hijacking by cancer cells, the possibility of reverse transfer, from cancer cells to NK cells, remains a completely open and promising new direction for research into tumor immune evasion and NK cell‑based immunotherapy.

MDSCs, a heterogeneous population of immature myeloid cells, are increasingly recognized as key participants in mitochondrial transfer networks that suppress antitumor immunity. Senescent tumor cells release mtDNA-containing EVs, which are selectively taken up by polymorphonuclear MDSCs (PMN-MDSCs), activating the cGAS-STING-NF-κB pathway and enhancing their immunosuppressive activity. Inhibition of mtDNA release through VDAC blockade reduces extracellular mtDNA levels, reverses PMN-MDSC-mediated immunosuppression, and improves chemotherapy efficacy in preclinical prostate cancer models [82]. In addition, macrophages can directly transfer intact mitochondria to granulocytic MDSCs (G-MDSCs) via TNTs, further enhancing their T cell suppressive function while reducing TNFα and IL-6 production [83]. Functional studies demonstrated that this mitochondrial transfer is required for G-MDSC immunosuppressive activity, as transfer of nonfunctional mitochondria failed to support disease progression in a mouse infection model [83]. These findings suggest that MDSCs exploit acquired mitochondria or mtDNA to reinforce immunosuppression, highlighting mitochondrial transfer pathways as potential therapeutic targets for reprogramming the tumor microenvironment.

### IMT among tumor, CAFs and endothelial cells

CAFs are co-opted by tumors to act as metabolic donors, with the voluntary or involuntary transfer of functional mitochondria being a critical mechanism of support. CAFs undergo aerobic glycolysis, producing high-energy metabolites like lactate and pyruvate. These metabolites are then imported by cancer cells and efficiently utilized in the TCA cycle within the acquired CAF mitochondria, fueling robust OXPHOS for energy production and biomass synthesis [84, 85]. CAFs promote metabolic crosstalk with tumor cells through direct mitochondria transfer via TNTs and via CAF-derived EVs [9, 84, 86]. In breast cancer, studies have documented TNT-mediated mitochondrial transfer from CAFs to malignant cells [9], whereas in lung cancer, CAF-derived EVs facilitate the transfer of mitochondria and mtDNA, enhancing cancer cell oxidative metabolism and metastatic potential [87].

Notably, endothelial cells have been shown to preferentially donate mitochondria to hypoxic tumor cells [88], providing a critical survival advantage to cancer cells experiencing metabolic stress. Experimental evidence further demonstrates that, following IMT, MCF-7 cells exhibit markedly increased resistance to doxorubicin. This enhanced chemoresistance is attributed to doxorubicin’s reliance on p53-mediated apoptotic signaling, whereas the transferred mitochondrial membrane proteins help preserve mitochondrial integrity and suppress apoptosis [88]. In addition, IMT from fibroblasts to cancer cells has been shown to rescue OXPHOS-deficient tumor cells from apoptosis [89], promoting chemoresistance and improving cancer cell survival in nutrient- or oxygen-limited microenvironments [88]. Furthermore, transferred endothelial mitochondria activate the Nrf2/HO‑1 pathway in melanoma cells, promoting tumor growth and polarizing macrophages toward an immunosuppressive M2‑like phenotype [13]. Despite these insights, the mechanisms and functional consequences of mitochondrial transfer from cancer cells to endothelial cells remain poorly defined. Clarifying this reverse pathway is essential for achieving a comprehensive understanding of metabolic symbiosis within the tumor microenvironment. Nonetheless, tumor-derived EVs are known to promote angiogenesis by directly or indirectly inducing endothelial activation through cytokines such as CCL18 [90]. This EV-mediated signaling is a key driver of pre-metastatic niche formation, ensuring a sustained supply of nutrients and oxygen necessary for the survival and expansion of disseminating tumor cells [91]. Within this context, it is plausible that mitochondrial transfer from tumor cells to endothelial cells occurs during metastasis and contributes functionally to disease progression.

## Functional consequences of mitochondrial transfer in cancer

### Metabolic reprogramming in mitochondria

OXPHOS supports anabolic metabolism by generating ATP through the ETC [92]. While early observations of upregulated glycolysis in oxygen‑rich environments led to the prevailing assumption that tumors rely primarily on glycolysis, accumulating evidence indicates that OXPHOS is active and competes with glycolysis in many tumor types, particularly in cancer stem cells [93]. For example, in pancreatic ductal adenocarcinoma (PDAC), forcing cells to depend on OXPHOS enhances invasive CSC phenotypes [94]. Similarly, in acute myeloid leukemia (AML), leukemic stem cells and resistant clones are highly dependent on OXPHOS, and MLL/AF9-mutated AML cells are especially sensitive to OXPHOS inhibition [95]. Pancreatic cancer cells also show strong reliance on mitochondrial respiration, and pharmacologic OXPHOS inhibition significantly suppresses tumorigenic traits [96]. Collectively, these findings demonstrate that OXPHOS activity is positively correlated with tumor aggressiveness and adaptive capacity. Thus, restoring OXPHOS through the acquisition of healthy mitochondria via IMT could rescue nutrient-deprived tumor cells and enhance survival under metabolic stress. This concept is reinforced by evidence that OXPHOS upregulation promotes tumor growth and adaptation to hypoxia, positioning OXPHOS as a key survival pathway under mitochondrial stress [93]. Collectively, IMT has been shown to function as a compensatory mechanism that restores bioenergetics, potentially enabling cancer cells to sustain growth under conditions of metabolic stress [97, 98].

Metabolic plasticity allows cancer stem cells to switch between different energy-producing mechanisms, such as OXPHOS and glycolysis. Recent studies have shown that IMT from neurons to cancer cells enhances metabolic plasticity, increasing the ability of cells to flexibly utilize and switch between OXPHOS and glycolysis [54, 99]. This enhancement may be mediated in part through the SIRT1/PGC-1α axis, a signaling pathway involved in mitochondrial remodeling that has been linked to cell stemness and plasticity [100]. SIRT1 is associated with invasion, proliferation, and cell stress of cancer cells. PGC-1α can mediate mitochondrial biogenesis and is activated by SIRT1. Together, these pathways can increase mitochondrial quantity and quality, leading to enhanced metabolic plasticity and cell stemness. This was further demonstrated in prostate cancer, in which CAFs donate mitochondria that activate the SIRT1/PGC-1α pathway, promoting mitochondrial remodeling and increased cancer stemness [86].

### Therapy resistance

#### Enhanced survival following chemotherapy, radiation, or targeted therapy

Therapies including chemotherapy, radiation, and targeted inhibitors are designed to induce mitochondrial damage and trigger apoptosis, theoretically leading to cancer cell death. However, tumor cells develop multiple adaptive mechanisms that minimize treatment-induced cytotoxicity. One major strategy involves the maintenance of persister and dormant cell populations. These rare cells survive initial therapy by entering a low-metabolic, non-proliferative state, rendering them intrinsically resistant to chemotherapeutic and radiotherapeutic agents. Mitochondrial injury during treatment can activate ATF4-dependent stress responses that promote the emergence of these persister/dormant cells, which later drive relapse and metastasis [101, 102]. Cancer cells also exhibit altered mitochondrial dynamics, modifying fusion and fission processes to withstand therapeutic stress [103, 104]. Through mitochondrial transfer, tumor cells can remodel their mitochondrial network, dilute damaged organelles, sustain ATP production, and evade apoptosis. Another mechanism of resistance involves drug detoxification. In chemoresistant cells, ABC transporters preferentially rely on mitochondrial-derived ATP, rather than glycolytic ATP, to power drug efflux. Thus, IMT indirectly supports drug resistance by maintaining mitochondrial fitness and enabling efficient drug export [105]. Additionally, regulators such as MCJ/DNAJC15 modulate mitochondrial respiration and influence ABC transporter activity, further reinforcing therapy resistance.

#### Targeted therapy evasion

KRAS encodes a small GTPase that functions as a key transducer in regulating cell proliferation [106]. Oncogenic KRAS mutations lock the protein in an active, GTP-bound state, driving uncontrolled proliferative signaling and increasing mitochondrial metabolic demands, including elevated ROS production and heightened OXPHOS dependency [107]. Consequently, IMT may provide a metabolic advantage that supports KRAS-driven tumor growth and contributes to therapeutic resistance. Indeed, KRAS-mutant tumors exhibit substantial reliance on mitochondrial metabolism; KRAS activation enhances mitochondrial ROS generation, which is required for tumorigenicity [108]. Disrupting mitochondrial metabolism, such as through inhibition of mitochondrial transcription factors like TFAM, has been shown to suppress KRAS-mediated cell growth, highlighting an opportunity to indirectly impair IMT-supported survival pathways. Resistance to MEK inhibition in KRAS-mutant tumors frequently arises through compensatory feedback that restores MAPK pathway activity [109]. Because the RAS/MAPK cascade proceeds from KRAS to RAF, MEK, and ERK, inhibiting MEK or ERK often triggers reactivation loops that sustain pathway signaling. Therapeutic strategies that combine inhibitors of mitochondrial transfer with MEK blockade may therefore be more effective, simultaneously disrupting IMT-enabled metabolic resilience and overcoming MAPK feedback-driven resistance.

### Immune modulation

#### Suppression or enhancement of antitumor immunity

Mitochondrial quality is closely tied to T cell function. Dysfunctional mitochondria can no longer support memory T cell formation, thereby reducing responsiveness and marking entry into an exhausted state. T cell dysfunction and exhaustion are hallmarks of the TME, and PD-1 blockade can partially reverse exhaustion [110]. However, IFN‑γ is required for this reversal, and mtDNA stress can adjust PD‑L1 expression via cGAS‑STING signaling, negatively affecting immunotherapy efficacy [111–113]. Therefore, even if IFN‑γ is present, transfer of stressed mtDNA from tumor cells may counteract the reversal of T cell exhaustion, suppressing anti‑tumor immunity. Homoplasmic replacement of T cell mitochondria by tumor‑derived organelles may thus represent one mechanism by which tumors limit the clinical benefit of PD‑1 blockade.

Importantly, the intersection of IMT with established T cell exhaustion programs extends beyond bioenergetics. The transfer of dysfunctional mitochondria activates the integrated stress response (ISR) and the transcription factor ATF4, which drives expression of exhaustion‑associated genes, including Tox and Pdcd1, while repressing effector molecules such as GzmB and Ifng [114, 115]. Concurrently, the accumulation of mitochondrial ROS sustains HIF‑1α activity, which promotes a metabolic switch toward glycolysis and further reinforces the epigenetic silencing of effector loci through increased DNA methyltransferase activity [64, 116]. Thus, IMT functions as a critical amplifier of canonical exhaustion pathways, and its blockade may synergize with immune checkpoint inhibitors by preventing the mitochondrial‑driven epigenetic lock that underlies T cell unresponsiveness.

#### Indirect immune modulation through stromal and metabolic networks

The immunomodulatory consequences of IMT extend beyond the direct donor-recipient pair, propagating through complex stromal networks that indirectly shape anti-tumor immunity. A paradigm-shifting concept is that cancer cells act as mitochondrial redistribution hubs that orchestrate the immunosuppressive ecosystem. Upon acquiring host mitochondria, cancer cells fuse and refurbish these organelles before preferentially redistributing them to immunosuppressive populations, including Tregs, TAMs and neutrophils, while simultaneously depleting CD8^+^ T cells [53]. This selective redistribution actively reconfigures the immune landscape by reinforcing the metabolic fitness of pro-tumor immune subsets at the expense of cytotoxic effectors.

Indirect immunosuppression also occurs via horizontal transfer of mitochondrial components to stromal fibroblasts. Cancer cells can transfer mutant mtDNA to CAFs via EVs, reprogramming CAFs toward an activated, pro-inflammatory phenotype characterized by increased secretion of immunosuppressive cytokines such as TGF-β and IL-6, as well as lactate accumulation. These CAF-derived factors, in turn, suppress CD8 + T cell proliferation and promote the expansion of Tregs, establishing a mitochondrial-driven, indirect immunosuppressive axis [117].

The TME further integrates inputs from non-immune stromal components, including the nervous system. Recent evidence demonstrates that neurons can transfer functional mitochondria to cancer cells, enhancing their metabolic plasticity and invasive capacity [118]. This neuron-cancer metabolic coupling may indirectly compromise anti-tumor immunity by promoting tumor progression and by modulating neurotransmitter release that influences immune cell function. Collectively, these findings indicate that IMT modulates immune function through a multi-tiered network involving cancer cells, immune subsets, fibroblasts, and neurons, rather than solely through direct mitochondrial exchange between cancer cells and effector T cells.

By integrating with these established metabolic and transcriptional programs, IMT not only directly impairs T cell function but also reinforces an epigenetic state of exhaustion that is maintained even after the removal of the initial stress. This multi‑layered impact, spanning bioenergetics, transcription factor networks, and chromatin remodeling, positions IMT as a central hub that links tumor metabolism to T cell fate determination (Table 3).

**Table 3:** Potential therapeutic interventions targeting mitochondrial transfer

Drug/Agent	Target site	Mechanism	Developmental stage	Ref
Cytochalasin B	Actin cytoskeleton	Inhibits actin polymerization and destabilizes existing TNTs	Only for research	[10, 57], 119– [121]
Cytochalasin D	Actin	Binds the hydrophobic cleft of actin monomers and prevents filament polymerization	Only for research	[29, 122, 123]
Latrunculin A/B	G-actin	Disrupts the actin cytoskeleton by inhibiting G-actin polymerization into F-actin	Only for research	[124–126]
Nocodazole	Tubulin	Disrupts microtubule assembly by interfering with α/β-tubulin polymerization	Only for research	[127, 128]
Vincristine	Tubulin	Inhibits tubulin polymerization and microtubule formation	An FDA‑approved chemo drug	[48]
GW4869	Neutral sphingo-myelinase 2	Inhibits EV biogenesis and release	Only for research	[129, 130]
Y-27,632	ROCK1/ ROCK2	Inhibits ROCK signaling to alter cytoskeletal dynamics and suppress EV release	Only for research	[131, 132]
Gap26	Connexin 43	Blocks gap junction and hemichannel communication between cells	Only for research	[133, 134]

## Methodological approaches for investigating IMT

**Fig. 3:**
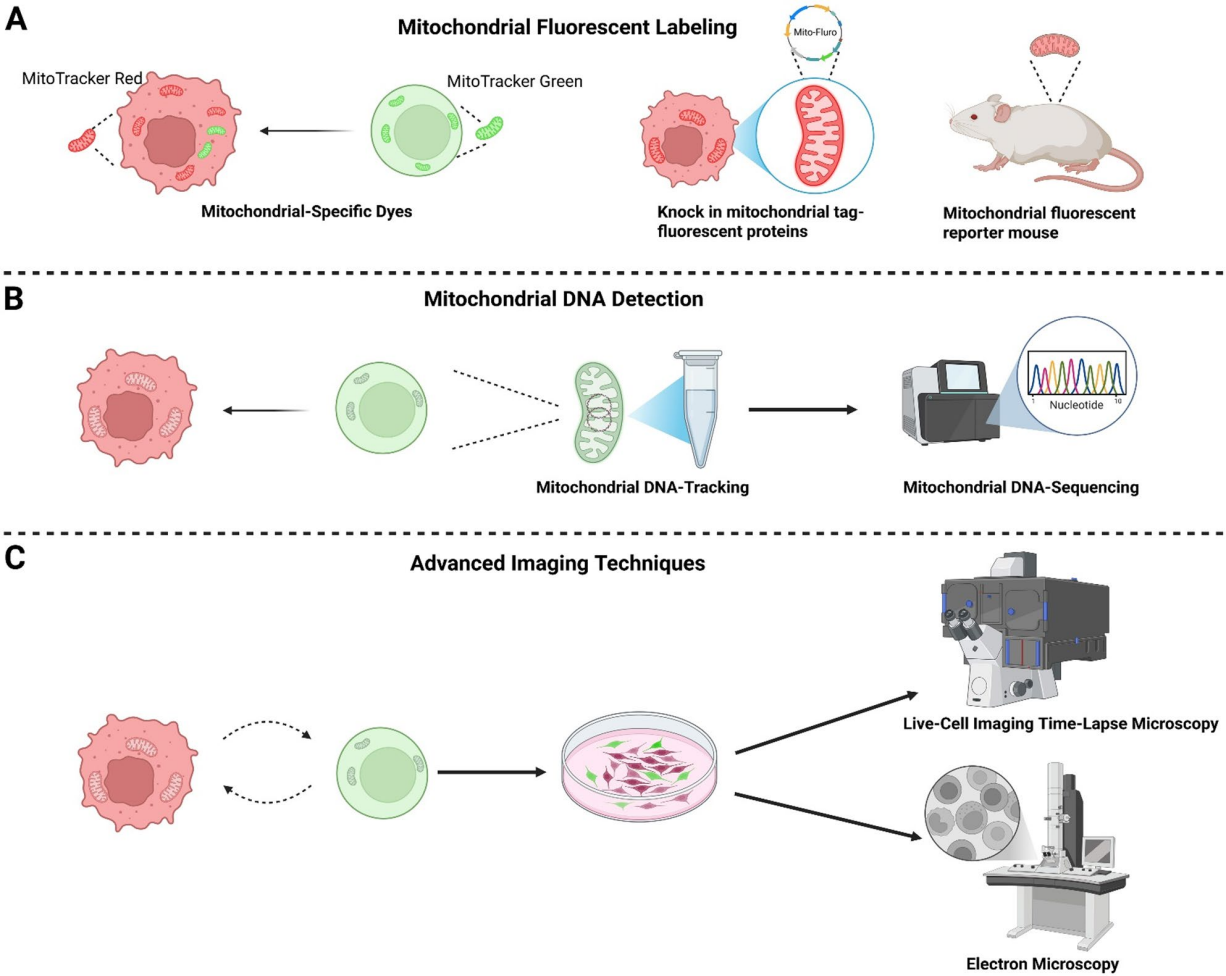
Methods for detecting and visualizing intercellular mitochondrial transfer. (**A**) Mitochondrial fluorescent labeling. Mitochondria in live cells can be labeled using specific dyes such as MitoTracker Red and MitoTracker Green. Transfer events are identified when both donor- and recipient-specific fluorescent signals are detected within the recipient cells, indicating acquisition of mitochondria. (**B**) mtDNA detection. The genetic material of transferred mitochondria can be traced by isolating and purifying mitochondria from recipient cells, followed by mtDNA sequencing to detect donor-specific mtDNA signatures. (**C**) Advanced imaging techniques. Real-time observation of mitochondrial transfer can be achieved using live-cell time-lapse microscopy to capture the kinetics of transfer. Electron microscopy can further confirm the physical presence and ultrastructural integrity of mitochondria within recipient cells. Figure created using Biorender (https://biorender.com)

Investigating IMT presents substantial methodological challenges, primarily due to the need to definitively distinguish transferred mitochondria from endogenous organelles and to capture dynamic transfer events within complex tumor microenvironments. Over the past decade, a number of genetic, imaging, and functional approaches have been developed and refined to address these challenges (Fig. 3).

### Genetic tracing

The most definitive approaches for tracking IMT rely on genetic markers that enable unambiguous identification of donor-derived mitochondria. mtDNA polymorphisms, naturally occurring variations in the mitochondrial genome, represent a powerful endogenous tracing system [135]. Because mtDNA is maternally inherited, present at high copy number, and accumulates variants at a relatively high rate, distinct mtDNA haplotypes frequently arise between individuals, between mouse strains, and even between tumor subclones. By leveraging single nucleotide polymorphisms (SNPs) or short insertion–deletion polymorphisms that differ between donor and recipient cells, researchers can quantitatively track mitochondrial transfer using a range of PCR-based assays or next-generation sequencing, circumventing the need for exogenous labeling completely [11, 136, 137]. Common formats include allele-specific quantitative PCR that targets a private donor SNP, high-resolution melting analysis of amplified mtDNA fragments, and digital droplet PCR that allows absolute quantification of donor-derived mitochondrial genomes in a mixed population. When coupled with deep sequencing of mtDNA, this approach can detect rare transfer events even when donor mitochondria represent a minor fraction of the total mitochondrial pool. This strategy was instrumental in the seminal discovery of horizontal mtDNA transfer in mouse tumor models, where the donor mtDNA haplotype was distinguished from that of the recipient cancer cells, and it remains a gold standard for validating IMT in vivo [137]. Its key strengths include complete avoidance of dye- or protein-based artifacts, compatibility with archival and clinical specimens, and the capacity to provide long-term lineage information. However, the resolving power depends on the genetic distance between donor and recipient; closely related populations with few mtDNA differences can be challenging to distinguish. Therefore, genetic tracing is most powerful when integrated with imaging and functional assays that provide complementary spatial and dynamic information.

### Functional assessment of transferred mitochondria

Demonstrating that transferred mitochondria provide functional benefits, rather than simply localizing within recipient cells, is critical for establishing their biological significance. The most commonly used approach is extracellular flux analysis, which measures oxygen consumption rate (OCR) as an indicator of oxidative phosphorylation and extracellular acidification rate (ECAR) as a measure of glycolytic activity. Comparing recipient cells before and after co-culture with mitochondrial donor cells enables quantification of the bioenergetic effects of mitochondrial transfer [138, 139]. These analyses are frequently complemented by stable isotope tracing (e.g., ¹³C-glucose or ¹³C-glutamine) combined with mass spectrometry to determine whether transferred mitochondria actively contribute to the TCA cycle and ETC.

Additional functional assays include ATP bioluminescence measurements, ΔΨm analysis using potentiometric dyes such as TMRM or JC-1, and redox-sensitive reporters (e.g., roGFP or HyPer) to monitor ROS dynamics. A major limitation of these approaches, however, is that they assess the combined activity of endogenous and transferred mitochondria, complicating attribution of metabolic improvements specifically to donor organelles. To overcome this challenge, studies commonly incorporate selective inhibitors of mitochondrial transfer and compare functional outcomes under transfer-permissive versus transfer-blocked conditions. The strongest evidence derives from rescue experiments in which a defined metabolic defect in recipient cells is restored by co-culture with wild-type donor cells, but not when mitochondrial transfer is physically inhibited.

### Imaging approaches for structural visualization

Electron microscopy techniques, including transmission electron microscopy (TEM) and focused ion beam scanning electron microscopy (FIB-SEM), provide ultrastructural evidence of TNTs and mitochondria within these intercellular connections [9]. Cryo-electron tomography (Cryo-ET) offers nanometer-scale three-dimensional visualization of TNT architecture and associated organelles in near-native states. However, it is important to note that while these techniques provide structural evidence consistent with IMT, they capture static snapshots and cannot definitively prove functional transfer. These methods are therefore most valuable when used in conjunction with genetic tracing or functional assays.

Fluorescence microscopy remains the most widely used method for real-time and endpoint visualization of intercellular mitochondrial transfer. MitoTracker dyes, including MitoTracker Green and MitoTracker Red CMXRos, are commonly used because they rapidly label mitochondria in live cells without requiring genetic manipulation. However, these dyes are associated with several important technical limitations. Because MitoTracker uptake is largely dependent on ΔΨm, depolarized mitochondria, which may still undergo transfer, can be inefficiently labeled or entirely missed [140]. Even dyes marketed as ΔΨm-independent may exhibit staining variability related to cellular redox status.

A major confounding issue is dye leakage. Lipophilic MitoTracker dyes can escape from pre-labeled donor cells into the extracellular medium and subsequently be taken up by recipient cells independently of true mitochondrial transfer, generating false-positive signals that may substantially overestimate transfer efficiency compared with genetically encoded mitochondrial reporters [138]. This artifact has been directly demonstrated in astrocyte–neuron systems, where conditioned medium alone was sufficient to transfer fluorescent dye signals in the absence of intact mitochondria [139]. Additional limitations include off-target accumulation within other membrane-bound organelles, such as the endoplasmic reticulum and lysosomes, as well as potential cytotoxicity and inhibition of ETC activity during live-cell imaging [140].

To overcome these challenges, genetically encoded fluorescent proteins targeted to mitochondria (e.g., Mito‑GFP, Mito‑DsRed, mito‑Keima) provide a more definitive and versatile alternative. Stable expression of distinct fluorophores in donor and recipient populations enables unambiguous identification of transferred mitochondria by confocal or super‑resolution microscopy [139, 141]. Furthermore, mito‑Keima, a pH-sensitive mitochondrial reporter, can distinguish transferred mitochondria based on changes in the recipient cell’s microenvironment, such as the pH shift accompanying entry into an acidic compartment [142]. Because these reporters are genetically encoded, they circumvent the leakage and membrane potential-dependent staining artifacts inherent to chemical dyes, and their stable expression allows for longitudinal tracking of mitochondrial fate following intercellular transfer.

### Omics approaches for characterizing consequences

Multi-omics approaches, including transcriptomics, proteomics, and metabolomics, do not directly measure IMT but are highly informative for defining its downstream consequences. Single-cell RNA sequencing (scRNA-seq) can capture transcriptional reprogramming in recipient cells following mitochondrial acquisition, while spatial metabolomics platforms such as desorption electrospray ionization mass spectrometry imaging (DESI-MSI) and matrix-assisted laser desorption/ionization mass spectrometry imaging (MALDI-MSI) enable label-free mapping of metabolite distributions across tumor tissues. A key limitation of these spatial approaches is insufficient resolution to reliably resolve metabolic signals at the single-cell level in highly heterogeneous tumors [143–145]. Emerging single-cell metabolomics technologies are beginning to overcome this barrier. Recent studies using atmospheric-pressure MALDI-MSI have achieved ~ 10 μm spatial resolution, enabling mapping of cell-type-specific metabolic states in glioblastoma and cancer–fibroblast co-culture systems [145, 146]. Moreover, integrative strategies that combine spatial metabolomics with single-cell transcriptomics and proteomics are poised to provide a more comprehensive view of how IMT shapes metabolic heterogeneity and cellular states within the TME [147, 148]. Continued advances in these technologies will be essential for resolving the metabolic consequences of mitochondrial transfer at cellular and ultimately subcellular resolution [51, 149].

## Therapeutic opportunities and challenges

**Fig. 4:**
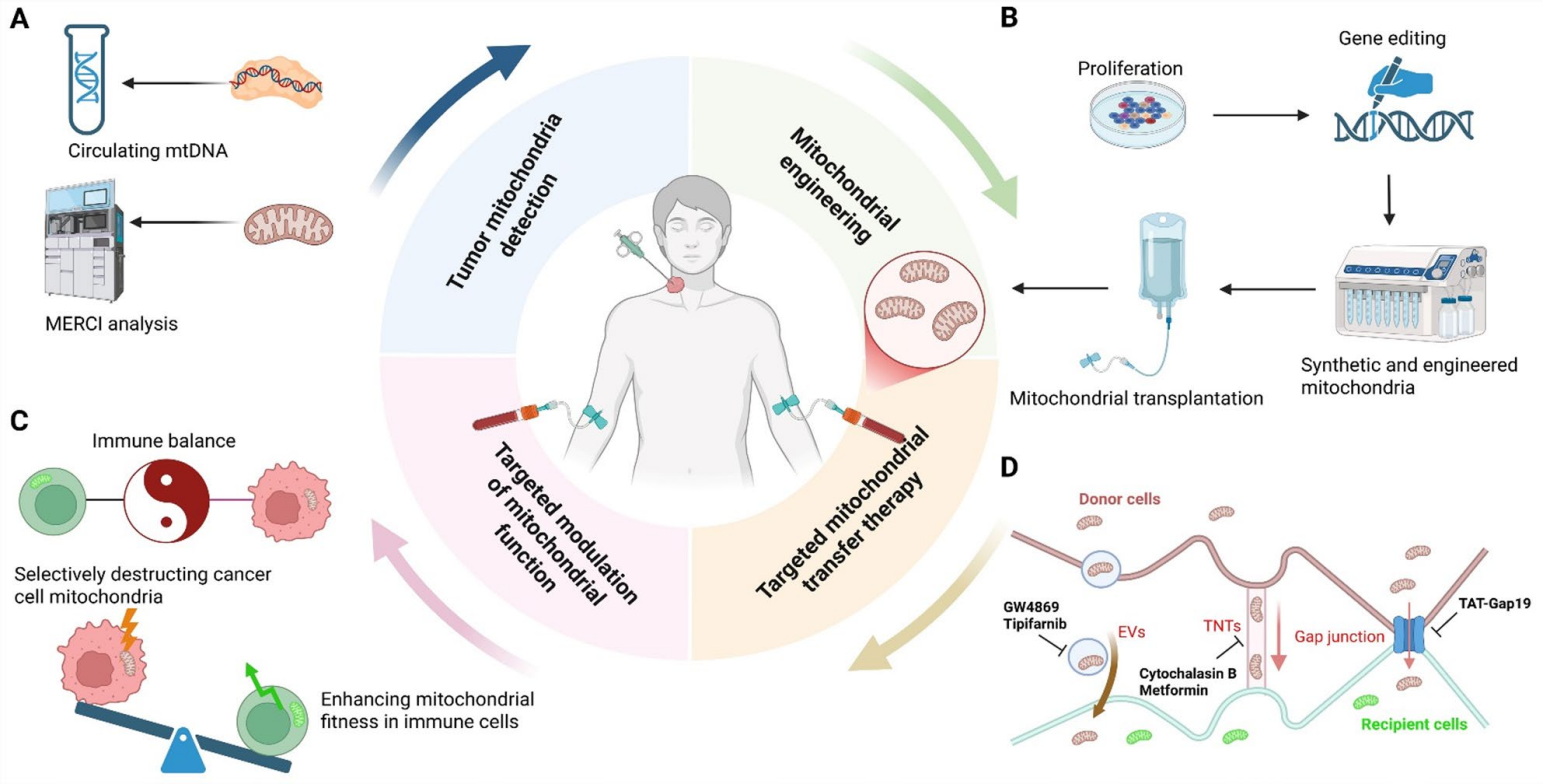
Mitochondria-targeted diagnostic and therapeutic strategies for intercellular mitochondrial transfer. (**A**) mtDNA in patient plasma serves as a dynamic, non-invasive biomarker for tumor burden and inflammatory status. Computational algorithms applied to scRNA-seq data can deconvolute the tumor microenvironment and infer intercellular mitochondrial transfer networks, identifying key donor and recipient cell populations. (**B**) Patient-derived mitochondria can be isolated, genetically edited (e.g., to correct mutations or introduce therapeutic transgenes), amplified, and re-delivered as autologous, engineered organelles to restore metabolic fitness in target tissues. (**C**) Dual modulation of mitochondrial function strategically rebalances the tumor microenvironment by enhancing OXPHOS and mitochondrial biogenesis in tumor-infiltrating immune cells to boost anti-tumor immunity, while simultaneously suppressing mitochondrial hijacking by tumor cells to limit proliferation and therapy resistance. (**D**) Pharmacological strategies aim to modulate mitochondrial transfer, including regulation of TNT formation, mitochondrial packaging into EVs, and control of motor proteins such as kinesin and dynein to direct organelle trafficking. Figure created using Biorender (https://biorender.com)

Understanding IMT and its progression into the TME holds promise for expanding therapeutic strategies (Fig. 4), but translating these insights into clinical interventions remains challenging due to measurement, context-dependence, and safety concerns.

### Targeting mitochondrial transfer mechanisms

Pharmacological inhibition of TNTs and EVs offers a direct strategy to disrupt mitochondrial transfer pathways that support tumor metabolism and therapeutic resistance [150]. Cytoskeletal inhibitors, including actin-targeting agents (cytochalasins, latrunculin A/B) and microtubule-disrupting drugs (nocodazole, vincristine), impair TNT formation by destabilizing the structural frameworks required for intercellular conduits, thereby limiting organelle transfer from stromal to tumor cells [10, 57, 119] (Table 3). In parallel, EV-mediated mitochondrial trafficking can be attenuated by targeting vesicle biogenesis pathways. Inhibition of neutral sphingomyelinase 2 with GW4869 reduces small EV release and alters vesicle composition, thereby disrupting mitochondrial cargo exchange between cells [151, 152] (Table 3). These interventions increase mitochondrial stress and potentiate apoptotic signaling, ultimately enhancing tumor sensitivity to chemotherapy and radiotherapy [120].

### Mitochondrial transplantation

Mitochondrial transplantation or mitochondrial therapy represents an emerging and promising concept in immunotherapy and regenerative medicine. Notably, studies in cardiac arrest models have demonstrated that mitochondrial transfer can mitigate tissue damage and improve functional recovery [153, 154]. Drug-loaded mitochondria have also been explored as targeted delivery vehicles to sensitize tumor cells. However, significant challenges remain, particularly in achieving tissue specificity and ensuring that engineered mitochondria are preferentially taken up by healthy cells rather than tumor cells [155, 156]. This is a critical concern because tumor cells can acquire mitochondria from surrounding stromal or immune cells. Engineered mitochondria have additionally been shown to enhance T-cell persistence in CAR-T therapies by improving mitochondrial metabolic fitness, ultimately boosting antitumor activity [157].

### Sensitization strategies

Sensitization strategies seek to integrate conduit blockade, mitochondrial transplantation, and immunotherapy into coordinated regimens that target complementary vulnerabilities in tumor–immune interactions. The central rationale is that tumors exploit bidirectional IMT to simultaneously acquire functional mitochondria from immune cells and export damaged organelles back to them, thereby promoting tumor fitness while impairing antitumor immunity [53]. A multipronged therapeutic framework can interrupt this pathological cycle at three distinct levels.

First, blockade of mitochondrial transfer conduits deprives cancer cells of stromal mitochondrial support required for resistance to chemotherapy and radiotherapy, while also preventing tumor-derived mitochondrial export that contributes to T cell dysfunction, thereby preserving immune competence [10, 57]. Second, mitochondrial transplantation can restore metabolic fitness in exhausted effector T cells by replenishing oxidative phosphorylation capacity and reducing ROS, thereby reversing exhaustion-associated programs and enhancing responsiveness to activating cues [42, 63]. Third, immune checkpoint inhibitors (e.g., anti-PD-1/PD-L1 antibodies) provide a final activating signal; however, their efficacy is often limited by underlying metabolic insufficiency. Restoration of mitochondrial fitness through the preceding interventions enables T cells to sustain proliferation and execute durable antitumor responses [158]. Although these approaches remain at a preclinical stage, their successful translation will require optimization of therapeutic sequencing, dosing strategies, and tumor-specific delivery systems.

### Challenges and perspectives

Although promising, sensitization approaches to IMT are tempered by key risks. Off-target disruption of normal cell communication could impair homeostasis [159, 160]. Engineered mitochondria raise concerns about immune rejection and adverse immune activation when combined with checkpoint inhibition [161, 162]. Additionally, tumor plasticity may drive metabolic switching and emergence of resistant clones, while genomic instability or mitochondrial mutations from manipulation could fuel secondary malignancies [163]. Cell-type-specific mitochondrial roles further complicate outcomes, highlighting the need for precise targeting and biomarkers [164].

Future IMT work should prioritize identifying the molecular cues that govern transfer specificity and regulation, addressing technical challenges in quantification and visualization within the TME, and developing tools (e.g., advanced reporters, high-resolution live imaging) to capture real-time transfer events and mitochondrial fate [7, 165]. Translational progress will require strategies that selectively disrupt harmful mitochondrial trafficking without compromising physiological transfer, exploration of mitochondrial transplantation to augment anti-tumor immunity, and the identification of biomarkers to guide patient stratification and inform combination therapies.

## Conclusions

IMT represents a bidirectional intercellular exchange in the TME that fuels cancer progression and therapy resistance. A growing body of evidence indicates that cancer cells exploit mitochondrial transfer pathways to gain a significant survival advantage. They can function as metabolic parasites by acquiring functional mitochondria from neighboring stromal cells, a process associated with meeting bioenergetic demands, promoting metastasis, and developing resistance to conventional therapies [98, 166]. Cancer cells siphon mitochondria from immune cells and stromal partners to enhance bioenergetics and metastatic capacity, while transferring damaged mitochondria to immune cells to suppress anti-tumor immunity. Therapeutic avenues, such as inhibiting TNT formation or blocking mitochondrial packaging into EVs, are under exploration, but require elucidation of regulatory cues and context-specific dependencies and the development of reliable biomarkers to guide clinical translation.

## Data Availability

No datasets were generated or analysed during the current study.

## Abbreviations

α-KGα: ketoglutarate
AML: Acute myeloid leukemia
BMSCs: Bone marrow stromal cells
CAFs: Cancer-associated fibroblasts
CTLs: Cytotoxic lymphocytes
Cryo-ET: Cryo-electron tomography
CSC: Cancer stem cell
CX43: Connexin 43
DAMPs: Damage-associated molecular patterns
DESI-MSI: Desorption electrospray ionization mass spectrometry imaging
ECs: Endothelial cells
EGFR: Epidermal growth factor receptor
ETC: Electron transport chain
EVs: Extracellular vesicles
G‑MDSCs: Granulocytic MDSCs
GSCs: Glioblastoma stem-like cells
HCC: Hepatocellular carcinoma
HMT: Horizontal mitochondrial transfer
IMT: Intercellular mitochondrial transfer
LC-MS/MS: Liquid chromatography mass spectrometry
LSCs: Leukemia stem cells
MALDI-MSI: Mmatrix-assisted laser desorption/ionization mass spectrometry imaging
MAPK: Mitogen-activated protein kinase
MDSCs: Myeloid‑derived suppressor cells
MDVs: Mitochondria-derived vesicles
mtDNA: mitochondrial DNA
MSCs: Mesenchymal stem cells
NK: Natural killer
OXPHOS: Oxidative phosphorylation
PDAC: Pancreatic ductal adenocarcinoma
PMN‑MDSCs: Polymorphonuclear MDSCs
ROS: Reactive oxygen species
scRNA-seq: Single-cell RNA sequencing
SNPs: Single nucleotide polymorphisms
TAMs: Tumor-associated macrophages
TILs: Tumor-infiltrating T cells
TME: Tumor microenvironment
TNTs: Tunneling nanotubes
TPP: Triphenylphosphonium
Tregs: Regulatory T cells
WGA: Wheat germ agglutinin
Δψm: Mitochondrial membrane potential
Δψp: Plasma membrane potential
